# Clinical factors related to successful or unsuccessful cardioversion in the EdoxabaN versus warfarin in subjectS UndeRgoing cardiovErsion of Atrial Fibrillation (ENSURE‐AF) randomized trial

**DOI:** 10.1002/joa3.12341

**Published:** 2020-04-15

**Authors:** Gregory Y. H. Lip, Jose L. Merino, Maciej Banach, Naab Al‐Saady, James Jin, Michael Melino, Shannon M. Winters, Monika Kozieł, Andreas Goette

**Affiliations:** ^1^ Liverpool Centre for Cardiovascular Science University of Liverpool and Liverpool Heart & Chest Hospital Liverpool UK; ^2^ Hospital Universitario La Paz Universidad Europea Madrid Spain; ^3^ Department of Hypertension Medical Univeristy of Lodz Lodz Poland; ^4^ Covance Inc. Osprey House Littlewick Green UK; ^5^ Daiichi Sankyo Pharma Development Basking Ridge NJ USA; ^6^ Daiichi Sankyo, Inc. Basking Ridge NJ USA; ^7^ Department of Cardiology, Congenital Heart Diseases and Electrotherapy Division of Medical Sciences in Zabrze Medical University of Silesia Zabrze Poland; ^8^ St Vincenz Hospital Paderborn Germany; ^9^ Working Group: Molecular Electrophysiology University Hospital Magdeburg Magdeburg Germany

**Keywords:** atrial fibrillation, cardioversion, edoxaban

## Abstract

**Background:**

EdoxabaN versus warfarin in subjectS UndeRgoing cardiovErsion of Atrial Fibrillation evaluated use of nonvitamin K antagonist oral anticoagulant edoxaban vs enoxaparin‐warfarin in patients with nonvalvular atrial fibrillation undergoing electrical cardioversion.

**Hypothesis:**

To assess clinical factors related to successful or unsuccessful cardioversion. To evaluate whether differences in adverse events based on anticoagulation strategy may exist.

**Methods:**

In this multicenter prospective randomized open‐label blinded end‐point evaluation trial, 2199 patients were randomized to edoxaban 60 mg once daily (30 mg for creatinine clearance 15‐50 mL/min, weight ≤ 60 kg, and/or concomitant use of P‐glycoprotein inhibitor) or enoxaparin‐warfarin. Successful cardioversion was confirmed by 12‐lead electrocardiography‐documented sinus rhythm.

**Results:**

Cardioversion was successful in 1578 patients; in 355 patients, cardioversion was unsuccessful. Male, high body weight, high body mass index (BMI), coronary artery disease, concomitant aspirin, or prior statins use were more common in patients with unsuccessful cardioversion; international normalized ratio control did not differ by cardioversion success. On multivariate analysis, gender (*P* < .05), body weight (*P* = .0196) and BMI (*P* = .0377) emerged as independent predictors of successful cardioversion. There were no significant differences in primary efficacy (a composite of stroke, systemic embolic event, myocardial infarction, and cardiovascular death during overall study period) regardless of cardioversion success. There were no significant differences in bleeding rates, regardless of cardioversion outcome; notwithstanding low numbers, edoxaban and enoxaparin‐warfarin did not differ.

**Conclusions:**

Male gender, higher mean weight and higher mean BMI were associated with unsuccessful cardioversion. Efficacy and safety outcomes were low and did not differ by cardioversion success.

## 1 INTRODUCTION

Successful cardioversion of atrial fibrillation (AF) depends on a number of clinical features, mostly reported from observational cohorts. Age, duration of prior AF, previous relapse, heart failure, and left atrial size are among the various factors that have been associated with unsuccessful cardioversion.^1^ Factors related to cardioversion procedure (ie, electrical vs pharmacological, paddle positions, as well as antiarrhythmic drug preloading) have also been investigated in relation to successful cardioversion.^1^ Nevertheless, there are relatively limited contemporary prospective data on thromboembolic and bleeding risks while on anticoagulation peri‐cardioversion, in relation to the success (or not) of the cardioversion procedure. The EdoxabaN versus warfarin in subjectS UndeRgoing cardiovErsion of Atrial Fibrillation (ENSURE‐AF) study (NCT 02072434) evaluated the use of the nonvitamin K antagonist oral anticoagulant (NOAC) edoxaban vs enoxaparin‐warfarin in patients with nonvalvular AF undergoing electrical cardioversion (ECV).^2^ Clinically relevant bleeding and thromboembolism rates were low and comparable between the treatment arms. Thus, ENSURE‐AF is the largest reported dataset of ECV of AF and a prospective multicenter trial, with adjudicated outcomes allowing us to determine the factors affecting successful cardioversion. The aim of this post hoc analysis from the ENSURE‐AF trial was to investigate clinical factors related to successful or unsuccessful cardioversion in a contemporary prospective clinical trial; and to report thromboembolic and bleeding risks on anticoagulation with warfarin or with a NOAC (edoxaban) in relation to successful or unsuccessful cardioversion.

## 2 METHODS

The design and principal results of the ENSURE‐AF trial (NCT 02072434) have been published.^2^, ^3^ In brief, this was a multicenter, prospective, randomized, open, blinded end‐point trial in patients with nonvalvular AF undergoing ECV that compared edoxaban 60 mg once daily (QD; 30 mg QD for creatinine clearance [CrCl] of 15‐50 mL/min, weight ≤ 60 kg, and/or concomitant use of P‐glycoprotein inhibitor) with enoxaparin‐warfarin in 2199 patients. Patients were stratified ie, according to cardioversion approach to transesophageal echocardiography (TEE)‐guided or non–TEE‐guided group. Patients with an international normalized ratio (INR) <2.0 at randomization received enoxaparin bridging and daily warfarin until the INR was ≥2.0 and those with INR ≥ 2.0 at the time of randomization did not require enoxaparin and were treated with warfarin alone; hence, edoxaban was compared to “optimized anticoagulation” with enoxaparin‐warfarin.^2^

In the TEE‐guided subgroup, TEE and ECV had to be performed within 3 days of randomization. In case of presence of thrombi on TEE, patients had an opportunity of completing 28 days of study medication without cardioversion or being discontinued from the study.

The primary efficacy endpoint defined in the main analysis was the composite of stroke, systemic embolic event, myocardial infarction, and cardiovascular death during the overall treatment period from randomization until end of study (28 days on study drug after cardioversion + 30 days follow‐up) and the primary safety endpoint was the composite of major and clinically relevant nonmajor bleeding during the on‐treatment period (time of first dose to last dose of study drug taken + 30 days). The trial protocol was approved by the ethics committees or institutional review boards in accordance with the Declaration of Helsinki. All patients provided written informed consent prior to participation in the study.

This post hoc analysis focuses on clinical factors related to successful or unsuccessful cardioversion in a contemporary prospective multicenter clinical trial; and reports thromboembolic and bleeding risks on anticoagulation in relation to successful or unsuccessful cardioversion.

Patients were followed for 28 days on study drug after cardioversion plus another 30 days to assess safety, which was analyzed in relation to success of cardioversion. Successful cardioversion was defined when the 12‐lead electrocardiogram prior to discharge showed sinus rhythm. Patients who had spontaneous cardioversion are considered as having successful cardioversion. Unsuccessful cardioversion was defined as AF on the ECG prior to discharge.

The number and per cent of patients with primary efficacy and safety outcomes were provided by treatment arm. Odds ratios and 95% confidence intervals are presented to assess the difference between treatment arms.

Baseline variables in which there are plausible differences (*P* ≤ .05) between successful and unsuccessful cardioversion patients are included as predictors of successful cardioversion in a logistic regression analysis. The trial primary analysis was based on intention‐to‐treat analysis so the patients with spontaneous cardioversion were also included in the main analysis. A sensitivity analysis of patients without spontaneous cardioversion was also performed. Because of likely correlations among predictors, a stepwise approach is applied. A significance level of .05 is required to allow a variable into the model and for a variable to stay in the model. The Hosmer‐Lemeshow goodness‐of‐fit test for the final selected model was performed.

## 3 RESULTS

Mean age ± SD was 64.3 ± 10 years in the edoxaban arm (n = 1095) and 64.2 ± 11 years in the enoxaparin‐warfarin arm (n = 1104). Baseline characteristics of patients are presented in Table 1. Cardioversion was successful in 1578 (81.6%) of those undergoing the procedure. Three hundred and fifty five (18.4%) patients had unsuccessful cardioversion. Of 2199 patients, 167 (7.6%) had spontaneous cardioversion.

**TABLE 1:** Demographic and baseline characteristics of patients prescribed edoxaban or warfarin

	Total (n = 2199)	Edoxaban (n = 1095)	Warfarin (n = 1104)
Age, mean (SD), y	64.2 (10.5)	64.3 (10.3)	64.2 (10.8)
Age ≥ 75 y, n (%)	359 (16.3)	172 (15.7)	187 (16.9)
Weight, mean (SD), kg	91.0 (18.7)	90.9 (18.3)	91.2 (18.7)
BMI, mean (SD), kg/m^2^	30.7 (5.7)	30.6 (5.6)	30.7 (5.8)
Gender, male, n (%)	1443 (65.6)	721 (65.8)	722 (65.4)
Geographical region, n (%)			
Eastern Europe	1299 (59.1)	650 (59.4)	649 (58.8)
Middle East and North Africa	82 (3.7)	39 (3.6)	43 (3.9)
North America	95 (4.3)	46 (4.2)	49 (4.4)
Western Europe	723 (32.9)	360 (32.9)	363 (32.9)
Type of AF, n (%)			
Paroxysmal (≤7 d)	415 (18.9)	208 (19.0)	207 (18.8)
Persistent (>7 d, <1 y)	1777 (80.8)	887 (81.0)	890 (80.6)
Medical history, n (%)			
Congestive heart failure	960 (43.7)	476 (43.5)	484 (43.8)
Coronary artery disease	378 (17.2)	181 (16.5)	197 (17.8)
Hypertension	1714 (77.9)	850 (77.6)	864 (78.3)
Diabetes mellitus	415 (18.9)	218 (19.9)	197 (17.8)
Peripheral artery disease	94 (4.3)	40 (3.7)	54 (4.9)
Renal impairment	250 (11.4)	129 (11.8)	121 (11.0)
ICH	5 (0.2)	2 (0.2)	3 (0.3)
Ischemic stroke/TIA	134 (6.1)	68 (6.2)	66 (6.0)
Anemia	85 (3.9)	42 (3.8)	43 (3.9)
Prior life‐threatening bleed	6 (0.3)	3 (0.3)	3 (0.3)
Prior active bleed	29 (1.3)	11 (1.0)	18 (1.6)
Creatinine clearance, mean (SD), mL/min	94.0 (35.2)	94.0 (35.7)	94.1 (34.7)
CHA_2_DS_2_‐VASc score, mean (SD)	2.6 (1.4)	2.6 (1.5)	2.6 (1.4)
CHA_2_DS_2_‐VASc score ≥ 2, n (%)	1707 (77.6)	841 (76.8)	866 (78.4)
HAS‐BLED risk score, mean (SD)	0.9 (0.8)	0.9 (0.8)	0.9 (0.8)
HAS‐BLED risk score ≥ 3, n (%)	59 (2.7)	26 (2.4)	33 (3.0)
Prior cardioversion, n (%)	595 (27.1)	291 (26.6)	304 (27.5)
Medications, n (%)			
Prior OAC	1599 (72.7)	791 (72.2)	808 (73.2)
Prior VKA	145 (6.6)	72 (6.6)	73 (6.6)
VKA currently	1071 (48.7)	513 (46.8)	558 (50.5)
NOAC currently	305 (13.9)	157 (14.3)	148 (13.4)
Full dose of edoxaban	2014 (91.6)	1001 (91.4)	1013 (91.8)

Abbreviations: AF, atrial fibrillation, BMI, body mass index, CHA_2_DS_2_‐VASc, Congestive heart failure, Hypertension, Age ≥ 75 y (2 points), Diabetes mellitus, Stroke (2 points), Vascular disease, Age 65‐74 y, Sex category; HAS‐BLED, Hypertension, Abnormal renal/liver function, Stroke, Bleeding history or predisposition, Labile International Normalized Ratio, Elderly (>65 y), Drugs or alcohol concomitantly; ICH, intracerebral hemorrhage; NOAC, nonvitamin K oral anticoagulants; SD, standard deviation; TIA, transient ischemic attack.

At the follow‐up visit (on day 28), sinus rhythm was maintained in 508 (64.3%) of patients on edoxaban and in 521 (66.1%) of patients on enoxaparin‐warfarin. Of those with recurrent AF after successful cardioversion, AF recurrence occurred among 270 edoxaban‐treated patients (34.2%); with n = 136 and n = 134 in the transesophageal echocardiography (TEE) and non‐TEE strata, respectively, and 248 enoxaparin‐warfarin‐treated patients (31.5%; with n = 131 and n = 117 in the TEE and non‐TEE strata, respectively. Median time to AF recurrence was 38 days (interquartile range [12‐43]) in edoxaban patients, and 36 (13‐50) in enoxaparin‐warfarin patients.

During the treatment period, beta blockers were the most commonly used rate control drugs in edoxaban‐treated patients (81.1%; n = 865) and in enoxaparin‐warfarin‐treated patients (81.4%, n = 881). Amiodarone was the most widely prescribed rhythm control agent, in both in edoxaban (41.0%, n = 438) and enoxaparin‐warfarin (40.9%, n = 442) groups, see Table 2.

**TABLE 2:** AF management during treatment period in patients prescribed edoxaban or warfarin

	Total (n = 2149)	Edoxaban (n = 1067)	Warfarin (n = 1082)
Beta‐blockers, n (%)	1746 (81.2)	865 (81.1)	881 (81.4)
Calcium channel blockers, n (%)	476 (22.1)	237 (22.2)	239 (22.1)
Digoxin, n (%)	233 (10.8)	124 (11.6)	109 (10.1)
Digitoxin, n (%)	20 (0.9)	8 (0.7)	12 (1.1)
Amiodarone, n (%)	880 (40.9)	438 (41.0)	442 (40.9)
Disopyramide, n (%)	2 (0.1)	0 (0.0)	2 (0.2)
Dofetilide, n (%)	2 (0.1)	1 (0.1)	1 (0.1)
Dronedarone, n (%)	18 (0.8)	6 (0.6)	12 (1.1)
Ethacizine, n (%)	4 (0.2)	3 (0.3)	1 (0.1)
Flecainide, n (%)	85 (4.0)	39 (3.7)	46 (4.3)
Propafenone, n (%)	181 (8.4)	94 (8.8)	87 (8.0)
Vernakalant, n (%)	2 (0.1)	1 (0.1)	1 (0.1)

Abbreviation: AF, atrial fibrillation.

Patients with unsuccessful cardioversion were more likely to be male (*P* = .0007), have higher body weight (*P* = .0006), have coronary artery disease (*P* = .0365) and receive concomitant use of aspirin (*P* = .0119) or prior use of statins (defined as statin use between 30 days prior to randomization and the first date of study medication) (*P* = .0202). Among those on warfarin, INR control was similar between patients with successful or unsuccessful cardioversion, whether assessed as time to achieve therapeutic range or time in therapeutic range (Table 3).

**TABLE 3:** Outcomes by cardioversion success

	Cardioversion success		*P*‐value
	Yes (n = 1578)	No (n = 355)	
Age, y, mean (SD)	63.9 (10.5)	64.4 (10.4)	.4334
Male, n (%)	1011 (64.1)	261 (73.5)	.0007
Weight, kg, mean (SD)	90.7 (18.5)	94.4 (18.7)	.0006
≤60 kg, n (%)	38 (2.4)	5 (1.4)	.2480
Body mass index, kg/m^2^, mean (SD)	30.56 (5.71)	31.22 (5.45)	.0472
Anticoagulant experienced, n (%)			
Current^a^ VKA user	773 (49.0)	171 (48.2)	.7808
Current^a^ NOAC user	207 (13.1)	58 (16.3)	.1110
CrCl, mL/min, mean (SD)	94.2 (35.1)	98.2 (35.2)	.0583
Medical history, n (%)			
Congestive heart failure	686 (43.5)	155 (43.7)	.9482
Coronary artery disease	264 (16.7)	76 (21.4)	.0365
Hypertension	1246 (79.0)	279 (78.6)	.8776
Diabetes	288 (18.3)	73 (20.6)	.3124
Peripheral artery disease	61 (3.9)	16 (4.5)	.5766
Valvular heart disease	339 (21.5)	85 (23.9)	.3114
ICH	3 (0.2)	0 (0.0)	.4110
Ischemic stroke/TIA	94 (6.0)	24 (6.8)	.5677
Myocardial infarction	111 (7.0)	24 (6.8)	.8550
Life‐threatening bleed	3 (0.2)	1 (0.3)	.7316
CHA_2_DS_2_‐VASc score, mean (SD)	2.6 (1.4)	2.6 (1.4)	.9591
AF history, n (%)			
Paroxysmal (≤7 d)	305 (19.3)	57 (16.1)	.1534
Persistent (>7 d, <1 y)	1273 (80.7)	298 (83.9)	.1534
TtTR (d), mean (SD)	7.6 (5.1)	8.0 (5.8)	.4991
TiTR (% of time), mean (SD)	70.9 (27.2)	71.3 (26.4)	.8688
TTR^b^ (% of time), mean (SD)	61.0 (30.1)	59.0 (30.6)	.4575
Drug therapies, n (%)			
Aspirin	166 (10.5)	54 (15.2)	.0119
ACEI/ARB	1007 (63.8)	227 (63.9)	.9636
Beta blockers	1243 (78.8)	274 (77.2)	.5108
Statins	593 (37.6)	157 (44.2)	.0202
Amiodarone	390 (24.7)	84 (23.7)	.6770

Abbreviations: ACEI, angiotensin‐converting enzyme inhibitor; AF, atrial fibrillation; ARB, angiotensin II receptor blockers; CHA_2_DS_2_‐VASc, Congestive heart failure, Hypertension, Age ≥ 75 y (2 points), Diabetes mellitus, Stroke (2 points), Vascular disease, Age 65‐74 y, Sex category; CrCl, creatinine clearance; ICH, intracerebral hemorrhage; NOAC, nonvitamin K antagonist oral anticoagulant; SD, standard deviation; TIA, transient ischemic attack; TiTR, time in therapeutic range; TTR, time in therapeutic range; TtTR, time to achieve therapeutic range; VKA, vitamin K antagonists**.**

^a^ “Current” defined as using VKA or NOAC at randomization or within 30 d prior to randomization. Percentages are based on the numbers of anticoagulant experienced.

^b^ Rosendaal method.

Gender, body weight, body mass index (BMI), baseline CrCl, coronary artery disease history, concomitant use of aspirin, and prior use of statins (Table 3) were included as predictors of successful cardioversion in a stepwise logistic regression analysis. Two models of a stepwise logistic regression analyses were made. The first model includes predictors of gender (odds ratio [OR] 1.584, 95% confidence interval [CI] 1.211‐2.071, *P* = .0008), BMI (OR 0.978, 95% CI 0.958‐0.999, *P* = .0377), concomitant use of aspirin (OR 1.347, 95% CI 0.945‐1.920, *P* = .0997), and prior use of statins (OR 1.250, 95% CI 0.977‐1.599, *P* = .0758). Gender and BMI emerged as independent predictors of successful cardioversion. The *P*‐value for the Hosmer‐Lemeshow goodness‐of‐fit test is 0.9192, which indicates that there is no evidence of poor fit; however, there are limitations with a stepwise approach and the p‐values may be underestimated.

The second model includes predictors of gender (OR 1.412, 95% CI 1.068‐1.865, *P* = .0153), body weight (OR 0.992, 95% CI 0.986‐0.999, *P* = .0196), concomitant use of aspirin (OR 1.359, 95% CI 0.954‐1.937, *P* = .0896), and prior use of statins (OR 1.257, 95% CI 0.983‐1.607, *P* = .0679). Gender and body weight emerged as independent predictors of successful cardioversion. The *P*‐value for the Hosmer‐Lemeshow goodness‐of‐fit test is .6678, which indicates there is no evidence of poor fit.

There were no significant differences in primary efficacy regardless of cardioversion success. There were no significant differences in bleeding rates (Table 4) regardless of cardioversion outcome, and, notwithstanding the low numbers, no difference in the edoxaban arm compared with enoxaparin‐warfarin.

**TABLE 4:** Efficacy and safety endpoints

	Cardioversion success	
	Yes (n = 1578)	No (n = 355)
Primary efficacy endpoint: stroke, SEE, MI, CVD		
Edoxaban	3/790 (0.4)	1/189 (0.5)
Enoxaparin‐warfarin	7/788 (0.9)	2/166 (1.2)
OR (95% CI)	0.43 (0.07‐1.87)	0.44 (0.01‐8.47)
*P*‐value	.3401	.9027
Primary safety endpoint: major + CRNM bleeding		
Edoxaban	10/787 (1.3)	4/189 (2.1)
Enoxaparin‐warfarin	4/786 (0.5)	4/166 (2.4)
OR (95% CI)	2.5 (0.72‐11.03)	0.88 (0.16‐4.78)
*P*‐value	.1784	1.0000
Major bleeding		
Edoxaban	1/787 (0.13)	2/189 (1.1)
Enoxaparin‐warfarin	3/786 (0.38)	1/166 (0.6)
OR (95% CI)	0.33 (0.01‐4.15)	1.76 (0.09‐104.73)
*P*‐value	.6236	1.0000

Note

The primary efficacy endpoint was the independently adjudicated composite of stroke, SEEs, MI, and CVD occurring between randomization until the end of study in the ITT analysis set.

The primary safety endpoint was the independently adjudicated composite of major and CRNM bleeding, from the time of first administration of study drug to end of treatment + 3 d in the safety analysis set.

Abbreviations: CI, confidence interval; CRNM, clinically relevant nonmajor bleeding; CVD, cardiovascular death; ITT, intention‐to‐treat; MI, myocardial infarction; OR, odds ratio; SEE, systemic embolic event.

## 4 SENSITIVITY ANALYSIS EXCLUDING SPONTANEOUS CARDIOVERSION

For this sensitivity analysis, patients with spontaneous cardioversion were excluded. Patients with unsuccessful cardioversion were more likely to be male (*P* = .0029), have higher mean weight (*P* = .0031), have coronary artery disease (*P* = .0210) and receive concomitant use of aspirin (*P* = .0075) or prior use of lipid modifying agents (*P* = .0092, Table 5).

**TABLE 5:** Outcomes by Cardioversion Success in patients without spontaneous cardioversion

Obs	Parameter	Cardioversion successful	Cardioversion unsuccessful	*P*‐value
1	n	1411	355	
2	Age, mean(SD)	63.89 (10.55)	64.37 (10.39)	.4395
3	Male, n (%)	920 (65.2%)	261 (73.5%)	.0029
4	Age > 65, n (%)	656 (46.5%)	169 (47.6%)	.7069
5	Weight (kg), mean (SD)	91.13 (18.39)	94.38 (18.67)	.0031
6	Weight ≤ 60 kg, n (%)	29 (2.1%)	5 ( 1.4%)	.4270
7	BMI, mean (SD)	30.64 (5.66)	31.22 (5.45)	.0814
8	Current VKA user, n (%)	710 (50.3%)	171 (48.2%)	.4690
9	Current NOAC user, n (%)	192 (13.6%)	58 (16.3%)	.1871
10	CrCL, mean (SD)	94.90 (35.25)	98.20 (35.18)	.1254
11	CHA2DS2‐VASc, mean (SD)	2.60 (1.43)	2.60 (1.42)	.9667
12	AF‐Paroxysmal, n (%)	242 (17.2%)	57 (16.1%)	.6230
13	AF‐Persistent, n (%)	1169 (82.8%)	298 (83.9%)	.6230
14	Congestive heart failure, n (%)	619 (43.9%)	155 (43.7%)	.9438
15	Coronary artery disease, n (%)	229 (16.2%)	76 (21.4%)	.0210
16	Hypertension, n (%)	1115 (79.0%)	279 (78.6%)	.8589
17	Diabetes disease, n (%)	259 (18.4%)	73 (20.6%)	.3413
18	Peripheral arterial disease, n (%)	57 ( 4.0%)	16 ( 4.5%)	.6926
19	Valvular heart disease, n (%)	311 (22.0%)	85 (23.9%)	.4423
20	Vascular interventions, n (%)	103 ( 7.3%)	34 ( 9.6%)	.1516
21	Intracranial Bleeding, n (%)	2 (0.1%)	0 (0.0%)	.4779
22	Nonintracranial bleeding, n (%)	55 (3.9%)	7 (2.0%)	.0780
23	Ischemic/embolic stroke or transient ischemic attack, n (%)	87 (6.2%)	24 (6.8%)	.6798
24	MI, n (%)	94 (6.7%)	24 (6.8%)	.9470
25	Labile INR, n (%)	135 (9.6%)	41 (11.5%)	.2652
26	Renal disease, n (%)	149 (10.6%)	42 (11.8%)	.4906
27	Life‐threaten bleed, n (%)	3 (0.2%)	1 (0.3%)	.8067
28	Concomitant‐acetylsalicylic acid, n (%)	144 (10.2%)	54 (15.2%)	.0075
29	Prior‐lipid modifying agents, n (%)	518 (36.7%)	157 (44.2%)	.0092
30	Prior‐agents acting on the renin‐angiotensin system, n (%)	906 (64.2%)	227 (63.9%)	.9255
31	Prior‐beta blocking agents, n (%)	1124 (79.7%)	274 (77.2%)	.3044
32	Prior‐amiodarone, n (%)	349 (24.7%)	84 (23.7%)	.6747

Abbreviations: AF, atrial fibrillation, BMI, body mass index, CHA_2_DS_2_‐VASc, Congestive heart failure, Hypertension, Age ≥ 75 y (2 points), Diabetes mellitus, Stroke (2 points), Vascular disease, Age 65‐74 y, Sex category, CrCl, creatinine clearance, INR, International Normalised Ratio, MI, myocardial infarction, NOAC, nonvitamin K oral anticoagulants, SD, standard deviation, VKA, vitamin K antagonist.

Gender, body weight, BMI, coronary artery disease history, concomitant use of aspirin, and prior use of statins were included as predictors of successful cardioversion in a stepwise logistic regression analysis. Two models of a stepwise logistic regression analyses were made. The first model includes predictors of gender (OR 1.500, 95% CI 1.153‐1.950, *P* = .0025), BMI (OR 0.982, 95% CI 0.962‐1.002, *P* = .0830), concomitant use of aspirin (OR 1.431, 95% CI 1.013‐2.020, *P* = .0418), and prior use of statins (OR 1.294, 95% CI 1.017‐1.648, *P* = .0362). Gender, concomitant use of aspirin, and prior use of statins emerged as independent predictors of unsuccessful cardioversion. The p‐value for the Hosmer‐Lemeshow goodness‐of‐fit test is 0.8430, which indicates no evidence of poor fit; however, there are limitations with a stepwise approach and the p‐values may be underestimated.

The second model includes predictors of gender (OR 1. 356, 95% CI 1.034‐1.777, *P* = .0275), body weight (OR 0.993, 95% CI 0.987‐1.000, *P* = .0382), concomitant use of aspirin (OR 1.442, 95% CI 1.021‐2.034, *P* = .0374), and prior use of statins (OR 1.295, 95% CI 1.018‐1.647, *P* = .0356). Gender, concomitant use of aspirin, prior use of statins, and body weight emerged as independent predictors of unsuccessful cardioversion. The *P*‐value for the Hosmer‐Lemeshow goodness‐of‐fit test is .5506, which indicates no evidence of poor fit.

There were no significant differences in the primary efficacy outcome regardless of cardioversion success. There were no significant differences in bleeding rates regardless of cardioversion outcome; see Table 6.

**TABLE 6:** Efficacy and safety endpoints in patients without spontaneous cardioversion

	Cardioversion success	
	Yes (n = 1411)	No (n = 355)
Primary efficacy endpoint: stroke, SEE, MI, CVD		
Edoxaban	3/718(0.4)	1/189 (0.5)
Enoxaparin‐warfarin	7/693 (1.0)	2/166 (1.2)
OR (95% CI)	0.41 (0.07‐1.81)	0.44 (0.01‐8.47)
*P*‐value	.3137	.9027
Primary safety endpoint: major + CRNM bleeding		
Edoxaban	10/717 (1.4)	4/189 (2.1)
Enoxaparin‐warfarin	4/691 (0.6)	4/166 (2.4)
OR (95% CI)	2.4 (0.70‐10.66)	0.88 (0.16‐4.78)
*P*‐value	.2009	1.0000
Major bleeding		
Edoxaban	1/717 (0.14)	2/189 (1.1)
Enoxaparin‐warfarin	3/691 (0.43)	1/166 (0.6)
OR (95% CI)	0.32 (0.01‐4.00)	1.76 (0.09‐104.73)
*P*‐value	.5970	1.0000

Note

The primary efficacy endpoint was the independently adjudicated composite of stroke, SEEs, MI, and CVD occurring between randomization until the end of study in the ITT analysis set.

The primary safety endpoint was the independently adjudicated composite of major and CRNM bleeding, from the time of first administration of study drug to end of treatment + 3 d in the safety analysis set.

Abbreviations: AOR, odds ratio; ITT, intention‐to‐treat; SEE, systemic embolic event.

## 5 DISCUSSION

In this post hoc analysis from ENSURE‐AF, our principal findings are that factors associated with unsuccessful cardioversion were male gender, higher mean weight and higher BMI. Second, efficacy and safety outcomes were low, and were nonsignificantly different whether cardioversion was successful or not.

Predictors associated with successful cardioversion have been investigated, and include various clinical, laboratory, and imaging parameters that have been reported in observational cohorts of varying sizes.^1^, ^4^ The strength of this ancillary analysis is evident from its being the largest reported dataset of ECV of AF based on a prospective multicenter trial (ENSURE‐AF), with adjudicated outcomes, thus allowing us to determine the factors affecting successful cardioversion.

As expected, approximately one third of the patients had AF recurrence, at a median time of approximately 36‐38 days, despite the use of beta blockers (81%) and amiodarone (41%).

In our study, there was no difference in the incidence of amiodarone therapy between the successful cardioversion group and unsuccessful cardioversion group. In one study,^5^ amiodarone therapy was considered to be effective in maintaining sinus rhythm following ECV in patient with paroxysmal and/or persistent AF. The efficacy of amiodarone was not influenced by the duration of AF; however, there were only 55 patients enrolled to this study with mean duration of AF > 12 months, thus the study might have been underpowered. Moreover, data on mean weight and BMI were not available.

The strongest predictor of successful cardioversion in observational cohorts is the duration of prior AF,^4^ and in our trial, patients with AF > 12 months in duration were excluded. In addition, elderly age and comorbidities are other clinical factors associated with unsuccessful cardioversion. Age was nonsignificant, but our trial population was relatively young (mean age 64 years).

In our study, male gender was associated with unsuccessful cardioversion. Women were underrepresented in this study. Possibly men could be affected by additional factors associated with unsuccessful cardioversion, like older age, comorbidities and weight. There is evidence^6^ that women are less likely to be treated with cardioversion despite that they tend to be more symptomatic with AF. One of the reasons may be their higher risk of recurrence of AF after successful cardioversion when compared to men.^7^, ^8^

Of note, this study clearly shows that body weight predicts unsuccessful cardioversion. This might be because of altered electrical field strengths in this situation. In addition, epicardial fat might also be a factor, which might interfere with the success of ECV.^9^, ^10^, ^11^ Moreover, patients with a higher body weight may have higher energy requirements for successful ECV.^6^

The concomitant use of aspirin and prior use of statins were also selected as predictors of unsuccessful cardioversion in patients without spontaneous cardioversion, and these are likely to reflect the concomitant comorbidities such as vascular disease. The above mentioned drugs may also indicate sicker patients. Indeed, vascular disease is a risk factor for failure of cardioversion within 30 days of ECV^7^, ^12^; however, the available data are conflicting. In one meta‐analysis of randomized controlled trials, atorvastatin was not associated with reduction of the risk of AF recurrence in the secondary prevention subgroup of patients with ECV.^13^ Statin use has previously been associated with improved success of cardioversion and maintenance of sinus rhythm.^13^, ^14^ In this study, we did not find such an association—but paradoxically, the converse was seen.

Our study shows that higher mean BMI was associated with unsuccessful cardioversion. In the ancillary analysis of ENSURE‐AF^15^ the BMI < 30 kg/m^2^ group had a greater rate of cardioversion success than the BMI ≥ 30 kg/m^2^ group. Higher probability of unsuccessful cardioversion in obese patients may reflect associated concomitant diseases or greater body impedance.^16^, ^17^

We did not find any significant difference in efficacy and safety outcomes related to the anticoagulation arm, but this is limited by the modest size cohort, limited follow‐up, and relatively lower risk. In any case, there is no significant difference between rate and rhythm control strategies, with regard to thromboembolic and bleeding outcomes.^18^

We had limited systematic data on echocardiographic parameters, as these were not systematically recorded. Moreover, precise data on the protocol of ECV were not available, as this was at investigator discretion (as ENSURE‐AF was an antithrombotic therapy trial, not a trial of a mandated cardioversion protocol). It was generally considered that the number and energy of ECV may influence efficacy of conversion to sinus rhythm in patients with paroxysmal and/or persistent AF. Patients who had recurrent cardioversion after relapse of AF, had sinus rhythm as long as or longer than after the first cardioversion.^16^

This was a post hoc ancillary analysis of a clinical trial to assess anticoagulation for ECV in AF, and any outcomes are underpowered and would simply be hypothesis‐generating. Nonetheless, this represents data from a contemporary randomized trial with adjudicated clinical outcomes.

To make our post hoc analysis clear, patients were divided into two groups according to maintaining sinus rhythm after cardioversion to evaluate clinical factors related to successful or unsuccessful cardioversion. However, the subgroup with patients without return to sinus rhythm and those who transiently returned to sinus rhythm appear to be heterogeneous.

In conclusion, in this post hoc analysis from ENSURE‐AF, our principal findings are that factors associated with unsuccessful cardioversion were male gender, higher mean weight and higher mean BMI. Second, efficacy and safety outcomes were low, and were nonsignificantly different whether cardioversion was successful or not.
